# Monitoring of Layered Thermoplastic Composites Using Shape Memory Alloys as Integrated Sensors for Multifunctional Lightweight Structures

**DOI:** 10.3390/ma18133193

**Published:** 2025-07-06

**Authors:** Michael Schwarz, Marius Weiler, Saravanan Palaniyappan, Steven Quirin, Maik Trautmann, Guntram Wagner, Hans-Georg Herrmann

**Affiliations:** 1 Chair for Lightweight Systems, Saarland University, 66123 Saarbrücken, Germanyhans-georg.herrmann@izfp.fraunhofer.de (H.-G.H.); 2 Fraunhofer Institute for Nondestructive Testing IZFP, Campus E3 1, 66123 Saarbrücken, Germany; 3 Group of Composites and Material Compounds (PVW), Institute of Materials Science and Engineering (IWW), Chemnitz University of Technology, 09125 Chemnitz, Germany; saravanan.palaniyappan@mb.tu-chemnitz.de (S.P.); maik.trautmann@mb.tu-chemnitz.de (M.T.); guntram.wagner@mb.tu-chemnitz.de (G.W.)

**Keywords:** GFRP composites, NDT, shape memory alloy, thermography

## Abstract

Since lightweight design and construction safety is a crucial element in different sectors of industry, the use of SMA wires in composites could improve the monitoring and adjustment of mechanical properties starting from the product development process through to field use. This work shows how embedded SMA wires can lead to a better understanding of applied forces to a composite structure made of GFRP laminates. To achieve this, different methods will be addressed. Besides mechanical testing of the GFRP-samples with embedded SMA wires, NDT-methods like active thermography, high-frequency ultrasonic testing, and computer tomography are used to detect the SMA wires, whereby thermography and computer tomography are best suited. In this study, the location and the amount of the applied force on GFRP composites with embedded SMA wires could be characterized with relative resistance changes. It is shown that SMA wires with a diameter of 250 µm are preferred to wires with a diameter of 100 µm due to production process and better performance under load (4N force plateau for 100 µm in contrast to 25N force plateau for 250 µm wires). Furthermore, Young’s modulus of the GFRP composites with embedded SMA wires was measured and is similar for various samples with 30.8 GPa on average.

## 1. Introduction

In aerospace, structural engineering, and robotics, lightweight design is an indispensable element, so smart materials and structures are becoming increasingly important. As a smart material, so-called shape memory alloys (SMAs) are used in various fields and applications, such as actuators in the automotive field [1], bone plates [2], and self-expandable stents [3], due to their significant actuation strain, recovery stress, and high actuation energy [4]. Due to the shape memory effect (SME) and superelasticity [4], SMAs are applicable in composite materials provided that they do not impair reinforcement, e.g., in the form of small-diameter wires. The principle of SME is as follows: a deformation in the martensite phase at low temperature can recover at high temperatures in the austenite phase. In case the recovery is blocked, SMAs experience significant actuation stresses up to 100 to 700 MPa. Compared to low-power hydraulic actuators (20 to 70 MPa) and piezoelectric actuators (1 to 9 MPa), these stresses are much higher [5]. In case of superelasticity, a relevant amount of strain can be recovered above austenite finish temperature.

In terms of material, copper-based, iron-based, and Ni-Ti-based SMAs are worth considering. Due to its advantages, like lower production costs, easier and safer handling, and superior mechanical properties [1], Ni-Ti-based SMAs are widely preferred in commercial applications. Another advantage is the possibility to additively manufacture little Ni-Ti-based SMA samples by laser powder fusion [6].

Besides these advantages, it is very challenging to integrate SMAs into polymer composites or to manufacture complex shaped components. It is crucial to verify where stiffer elements have to be placed to optimize the structure [7]. Like in every fiber reinforced composite, it is necessary to achieve a strong bond between the SMA wire and the matrix. This strength should be in a range where it is high enough for a robust structure but not too high, where cracks can propagate rapidly, leading to undetectable damage and potentially catastrophic failure [8]. As shown in [9], the surface roughness of the metal and the joining process temperature for the composite is very important for different metal–polymer systems. In [10], different coating treatments for SMA wires are conducted and tested. It is shown that chemical treated wires with an adhesion promoter coating have the highest adhesion strength. So, wires with the aforementioned treatment are used in this study. In contrast to [11], where SMA wires with a diameter of 600 µm and no chemical treatments are embedded with an indentation method in a GFRP composite or [12] where a CFRP composite with SMA wires of a diameter of 510 µm is used, in this study, a hand lay-up procedure with prepregs and wire diameters of 100 µm and 250 µm in a GFRP composite is used. Mechanical properties of SMA wires with different diameters (100 µm and 250 µm) and mechanical properties of composites with embedded SMA wires are valuated and combined to state which diameter of SMA wires are best suited for the GFRP composites.

The main focus of this study will be the indentation of a probe tip on GFRP composites with embedded SMA wires and monitoring the signals. We must investigate the information that SMA wires give about the location, amount, or distance of the indentation point.

Another focus in this study is the characterization of GFRP composites with SMA wires by non-destructive testing methods. In this paper, different non-destructive testing (NDT) methods like thermography, computer tomography, and ultrasonic are used and compared to detect the integrity, location and profile of the SMA wires and the composite.

## 2. Materials and Methods

### 2.1. Production of Samples

Samples of GFRP laminates (50% glass fiber) with embedded SMA wires were fabricated using hand lay-up procedure of prepregs. Prepregs consisting of thermoplastic PA6 are laid down with alternating orientation. The used SMA wires (*NiTi #1*, Fort Wayne Metals, Fort Wayne, IN, USA) consist of 54.5% to 57% mass nickel and balance titan, with less than 0.05% of carbon, cobalt, copper, chromium, hydrogen, iron, niobium, nitrogen, and oxygen (ASTM F2063-18) [13]. Nickel–titanium wires with a diameter of 100 µm and 250 μm were used in this study. The manufactured wires were straight annealed, providing a light oxide finish. Before every surface treatment procedure, the NiTi samples underwent thorough cleaning for 15 min each, utilizing ultrasonication in ethanol and deionized (DI) water, to meticulously remove contaminants such as grease, dust, foreign particles, etc.

In a differential scanning calorimetry (DSC), the following transformation temperatures were measured: austenite start temperature −0.56 °C, austenite finish temperature 21.85 °C, martensite start temperature 19.26 °C, and martensite finish temperature −3.95 °C.

**Figure 1:**
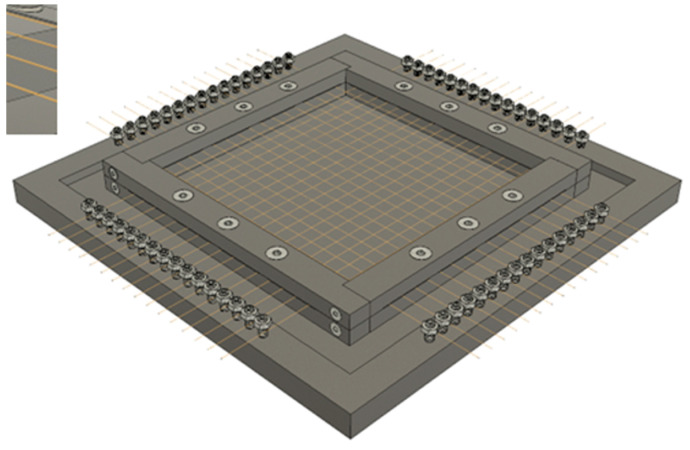
Custom rig for prestraining SMA wires during thermoforming.

**Figure 2:**
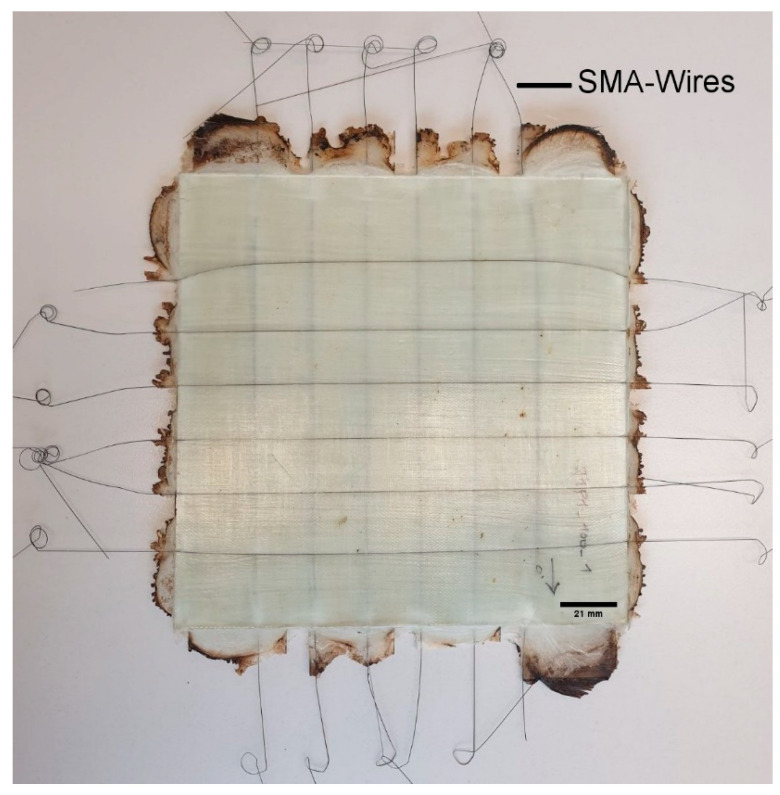
GFRP sample with incorporated and prestrained SMA wires.

Twelve superelastic SMA wires with a prestrain of 2% w1qere placed between the prepregs. Six wires in horizontal direction and six wires in vertical direction, to obtainachieve a layer configuration as follows: [0°, 90°, Wires (90°), 90°, 0°, 90°, 0°, 90°, 0°, Wires (0°), 0°, 90°]. The subsequent lamination process was controlled precisely with following parameters: temperature, duration and pressure. For keeping prestrain during the thermoforming of samples, a custom rig was built, as can be seen in Figure 1:

SMA wires in the laminate have a spacing of approximately 20 mm with the endings looking out of the laminate for a connection to a circuit board to measure change in resistance during force load. A finished sample is depicted in Figure 2. The reserve between a pressing force that keeps the press closed but does not break the wires can lead to a visible amount of overflow in the current state of the process. This overflow material will be removed before measurements.

**Figure 3:**
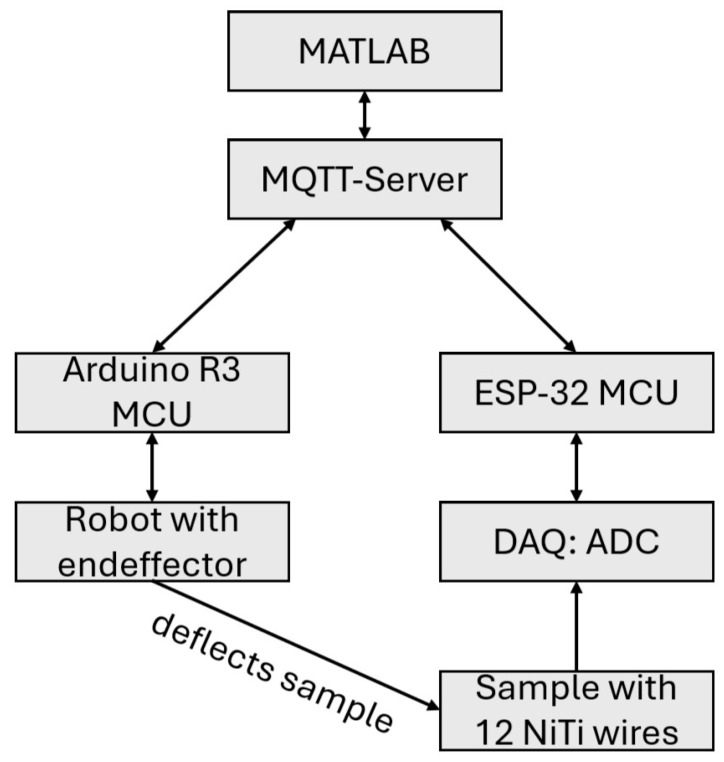
Scheme of test rig for applying point pressure on GFRP samples. Custom PCB with ADC for measuring change in resistance of SMA wires during sample deformation.

Figure 3 shows the scheme of the custom setup for applying point pressure on samples designed and built, as can be seen in Figure 2. A probe tip is installed parallel to z-axis with a force sensor, to monitor the applied force.

For the simultaneous recording of the resistance change in SMA wires, a custom printed circuit board was designed. As an analog to digital converter (ADC) an ADS1258, which has 16 input channels and 24-bit resolution, is used. Each channel has its own quarter-bridge with an amplifier circuit and gain of 1001. The SMA wire resistances were assumed 4.3 Ω and the quarter bridges were designed accordingly. ADC measurements were sent to an ESP-32, which is a micro control unit, and from there to a MQTT-server. A running MATLAB-script (version: R2025a) scrapped the data from the MQTT-server. The ADC takes approximately 1900 samples per second (SPS), while multiplexing through the twelve connected channels with SMA wires.

The testing of samples was performed using the rig with probe tip (see Figure 3). Samples were placed in the setup while being held at the corners, so they could be deformed with the probe tip. At programmed points, the probe tip presses into the sample from above with a constant velocity of 0.0492 mm/s until an applied weight force of 1500 g has been reached.

### 2.2. Non-Destructive Testing Methods

#### 2.2.1. Thermography

Thermography is an imaging non-destructive testing method for material testing where the surface temperature of samples or components is measured. The temperature is measured with an infrared camera. This is possible because every object with a temperature above 0 Kelvin sends out thermal radiation [14]. As the emitted radiation is a function of temperature it can be stated that with increasing temperature the intensity of radiation rises. When radiation strikes the surface of an object there are three possibilities: The radiation is reflected, absorbed or transmitted [15]. Depending on material properties, the level of reflection, absorption or transmission varies [16].

Non-destructive testing with active thermography is described with the norm DIN 54192 [17]. For active thermography, different excitation methods can be used, for example, flashlight, eddy current, or IR-arrays. With flashlight excited thermography, different defects like impact [18,19,20,21], delamination [22,23,24] or porosity [25] can be characterized in different materials like CFRP, metals or multimaterial composites. Additionally, a Fast Fourier transformation (FFT) can be applied to improve the results, as shown in [18,24,26,27]. In this paper, an excitation method is chosen where the wires are heated due to an electric current. The infrared camera used in this study is a VarioCAM HD head (InfraTec) and has an uncooled microbolometer Focal Plane Array (FPA)-detector with a resolution of 1024 × 768 pixel or 640 × 480 pixel. In this study a resolution of 1024 × 768 pixel was used. The spectral range is 7.5–14 µm, with a IR-image frequency of 30 Hz and temperature measuring range is between −40–1200 °C. The temperature resolution at 30 °C is better than 0.05 K and measuring accuracy is ±1.5 K or ±1.5%.

#### 2.2.2. Ultrasonic Testing

**Figure 4:**
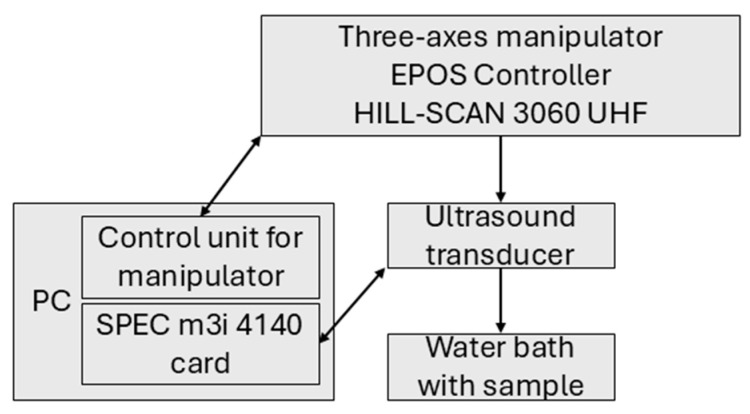
Scheme of ultrasonic testing.

The experiments with high-frequency ultrasonic testing are performed in a water bath. The focusing ultrasonic transducer (IAPF-F25.6.1, GE Sensing & Inspection Technologies GmbH, Pforzheim, Germany) is moved by a three-axes manipulator with an EPOS controller, a HILL-SCAN 3060 UHF (by Hillger, Braunschweig, Germany). Furthermore, it has a center frequency of 23.4 MHz (25 MHz) and is used in pulse-echo technique. A SPEC m3i 4140 card (by Spectrum Instrumentation GmbH, Grosshansdorf, Germany) connects the transducer to the PC. A scheme of the experimental setup is shown in Figure 4.

The transducer is placed at the surface of the water with a distance equal to its focal length of 24.6 mm. The transducer scans the surface of the sample in a defined area, the so-called region of interest (ROI). The maximum lateral resolution of the 25 MHz transducer is approximately 300 µm and the axial resolution 55 µm.

#### 2.2.3. Computer Tomography

In computer tomography testing, X-rays penetrate a sample, where the X-rays are damped. This damping occurs due to absorption of the X-rays in the sample and depends on the materials in the sample. The penetrated sample is depicted on a detector, so the result is a two dimensional radiograph. In computer tomography the sample is rotated 180 degrees and for different angles radiographs are recorded. These radiographs are reconstructed with a mathematical method to a digital volume picture. In this study the micro-CT scanning was performed using a scanner named CT-alpha (ProCon X-ray, Saarstedt, Germany), which was equipped with an X-ray tube Feinfocus FXT-160.51 (Comet X-ray, Flamatt, Switzerland) and an X-ray detector Shad-o-Box 6 K HS (Teledyne DALSA Waterloo, ON, Canada). A voltage of 120 kV and a current of 100 μA were configured to obtain high-contrast images with a high signal-to-noise ratio. To obtain a good resolution, the magnification was set to about ×1.1 resulting in a voxel size of 35 μm. A filter to reduce ring artifacts and beam hardening correction were applied to improve image quality. The detailed parameters of the CT scanning are given in Table 1.

## 3. Results

**Figure 5:**
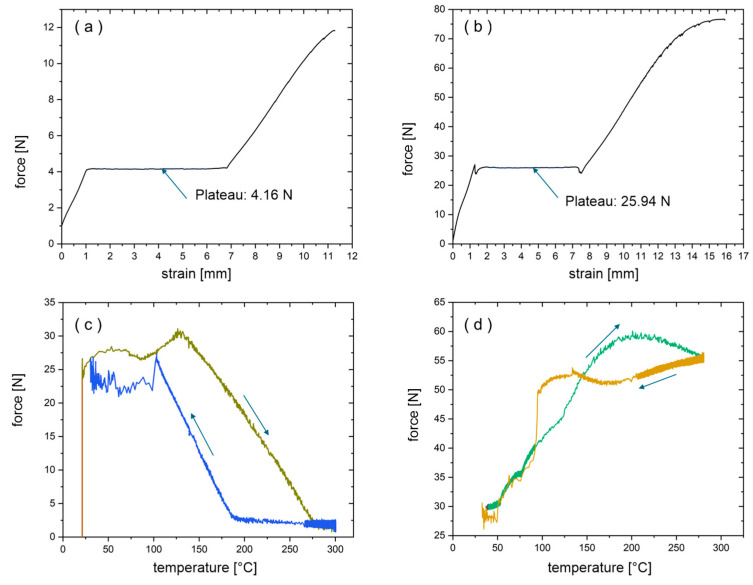
Strain-Force-Plots for (**a**) 100 µm and (**b**) 250 µm NiTi wire. Temperature–force Plots of a NiTi wire with 250 µm diameter with (**c**) Pre-force of 26 N (Plateau) and (**d**) Pre-strain of 4%.

In this paper tensile tests of SMA wires (100 µm and 250 µm in diameter) in their as-received state are measured to evaluate the relationship between strain, stress, and electrical resistance (Figure 5). In Figure 5a,b, it is shown that wires with a diameter of 100 µm only reach 16% of the force plateau than wires with a diameter of 250 µm.

Additionally, it is important to evaluate how the wires behave during the pressing process in laminate production. Pretests where conducted in a thermal chamber connected to an electrically controlled testing machine to analyze if the wires should be treated with a predefined preload (based on the plateau level from previous tests) or to a specified pre-strain.

Whether the wires undergo a preload to a defined force (plateau level) or a pre-strain to a defined elongation, the behavior under temperatures of 300 °C (pressing temperature) were significantly different. Figure 5c,d compare the temperature–force behavior for both cases. In case of a preload, the force increased slightly until a turning point of 130 °C, after this point the preload steadily decreased with rising temperature. The preload returns to its starting level after cooling to room temperature. In the case the wire is placed between the laminates, it can potentially move out of position at higher temperatures and freeze in a state of undefined strain. In the other case when the temperature ramp was applied after the wire was pre-strained to 4%, the preload increased steadily until 200 °C. When the wire cooled down to room temperature, the starting force level was regained, too.

In summary, in case of the insufficient pre-strain, the wire can lose its position during processing, whereas exorbitant tension may lead to wire breakage. Therefore, after these pretests we can conclude, that a strain-controlled preload of 2–4% is recommended for laminate production. Additionally, for the GFRP composites with SMA wires, wires with a diameter of 250 µm are used because of the better mechanical properties in comparison to wires with a diameter of 100 µm.

Twelve connected SMA wires incorporated in a GFRP were measured simultaneously. The measured resistances of all wires without sample deformation can be seen in Table 2. Resistance is measured in the unit Ω. A value in Table 1 which is (.) shows that the wire could not be measured due to a lack of connection or because the resistance is out of the measurement range.

**Figure 6:**
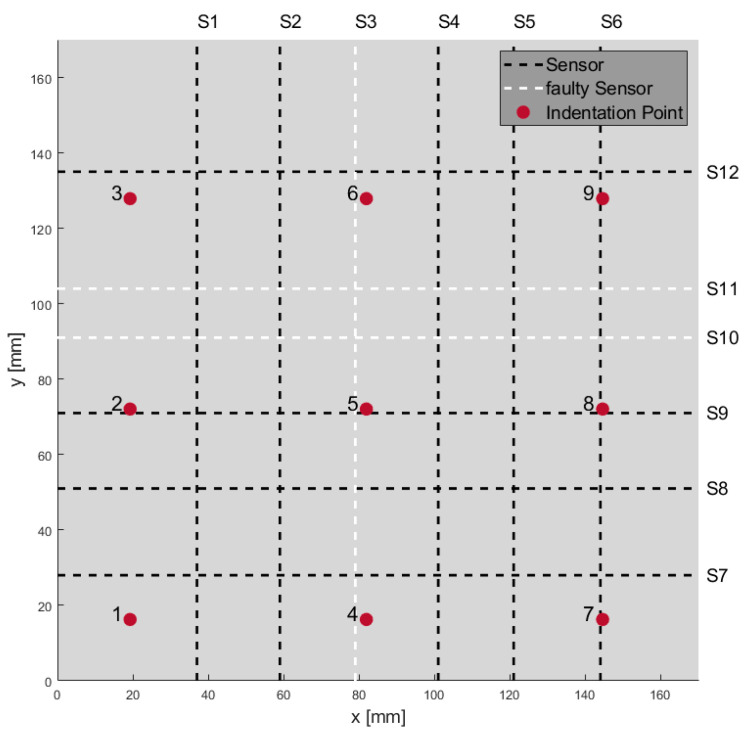
Scheme of a sample with twelve sensors and nine different indentation points.

**Figure 7:**
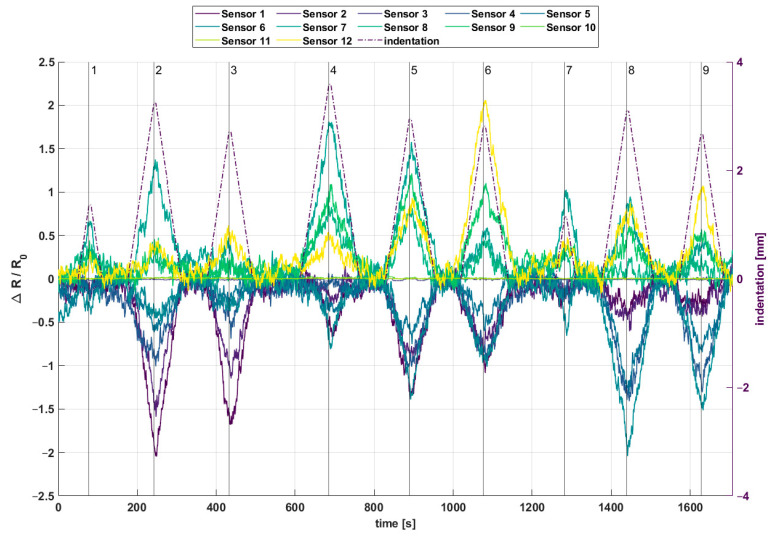
Nine different points of applied weight force causing change in resistance of wires.

From the initial 60 wires, 41 wires could be measured, whereby in sample 2 and 3 only six wires could be measured. In sample 1, four ten wires could be measured. There is no sample where all twelve wires could be measured. The resistance range of the wires are between 3.65 Ω und 4.55 Ω, with an average of 4.02 Ω. These measured resistances are the reference for the measurements with an applied force (see Figure 6 and Figure 7).

In Figure 6, a scheme of a sample with twelve wires (sensors) and nine different indentation points is shown. In Figure 7 the recorded signals of the twelve sensor channels are shown in a diagram. While force was applied with an indentation of up to 3.5 mm, relative change in resistance of 0.0004 to 0.0021 ΔR/R_0, depending on the distance between point of applied weight force and location of wires in samples, has been measured. The change in resistance also corresponded to layer position of the wires. Due to geometrical and production process reasons, wires (sensors) 1–6 are placed above the neutral axis and wires 7–12 are placed below the neutral axis. In case the wires are placed above the neutral axis the resistance decreased, because of the compression of the wires. If wires were below the neutral axis, resistance increased due to stretching of the wires. For example, at indentation points 4, 6, and 9, it can be seen that relative resistance change is the highest for wires near to these indentation points. For indentation point 4 which is near sensor S2, S4 and S7 relative resistance change in S7 (1.8 × 10^3^) is the highest followed by S4 (−0.75 × 10^3^) and S2 (−0.6 × 10^3^). At indentation point 6, which is near sensors S2, S4, S9 and S12 relative resistance change in S12 (2.1 × 10^3^) is the highest followed by S9 (1.1 × 10^3^), S2 (−1.1 × 10^3^) and S4 (−1 × 10^3^). At indentation point 9, which is near sensors S5, S6 and S12 relative resistance change in S6 (−1.5 × 10^3^) is the highest followed by S5 (−1.3 × 10^3^) and S12 (1.1 × 10^3^).

**Figure 8:**
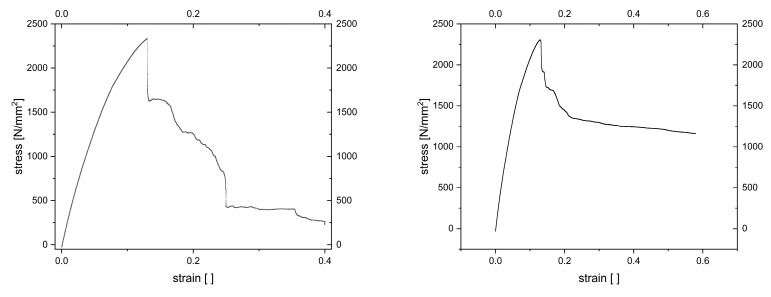
Stress–strain-diagrams of two SMA integrated GFRP composites during tensile test with a speed of 0.2 mm/s.

Besides indentation tests, there were tensile tests performed on GFRP laminates with integrated SMA with diameters of 250 µm. These tensile tests were conducted with a speed of 0.2 mm/s for multiple samples at room temperature with a subsequent calculation of the Young’s modulus. Figure 8 shows two diagrams of the tensile tests. In both diagrams, the stress rises to almost 2500 N/mm^2^ and then decreases abruptly due to failure. In the left diagram, the stress decreases in several steps to a very low level whereas in the right diagram the stress decreases constantly to a higher level than in the left diagram. These differences show the problems in the manufacturing process of the composites. The calculated Young’s modulus for the laminate in the left diagram is 32.5 GPa and for the laminate on the right is 29.4 GPa. Other laminates where tested too, thus the average for the Young’s modulus is approximately 30.8 GPa. This shows that despite all problems in production processing mechanical properties of the produced samples are similar.

**Figure 9:**
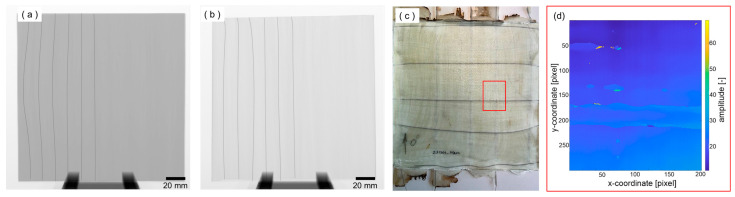
Computer tomography images of a sample with (**a**) additional aluminum cover layers on both sides and (**b**) without cover layers. Image of the sample and marked in red (**c**), where the ultrasonic measuring area is located. C-scan of the measuring area with runtime as color (**d**).

In addition to tensile tests, various non-destructive testing methods were explored with the aim of detecting wire positions, wire breaks, or defects in the laminate. Computer tomography, ultrasound, and thermography were investigated on preliminary samples. These also included a modification of the original sample having thin aluminum sheets as cover layers on both sides to investigate the influence of additional metallic cover on the NDT measurements. In computer tomography, no matter if the samples were without cover layers or with aluminum, the positions of the wires with a diameter of 250 µm were clearly visualized through X-ray imaging. It is clearly visible that wires are not broken but sound. An example is depicted in Figure 9a for a sample with an aluminum cover layer and Figure 9b without an aluminum cover layer. The determination of the wire position in the thickness direction was possible with computer tomography, too, due to rotating the sample around its own axis.

Ultrasonic testing measurements were performed using the immersion method with pulse-echo and a 25 MHz transducer, described in Section 2.2.2. Figure 9c,d show the characterized area of a SMA wire in the sample without aluminum cover layer (in red) and the corresponding C-Scan, respectively. During measurements, the polymer reinforcement caused significant signal attenuation and despite a theoretical lateral resolution of 300 µm SMA wires with a diameter of 250 µm were not clearly visible in the C-scan whether with or without aluminum cover layers. Nevertheless, there is an area in the C-Scan were a SMA wire can be assumed. This horizontal line is around pixel 200 and is 40 pixels thick (4 mm). This could be explained as an interphase of the SMA wire in the sample or as a delamination around the SMA wire. Other smaller defects (holes) in the upper part can be detected, as well. In comparison, computer tomography is able to detect the position and possible breaks of SMA wires whereas ultrasonic testing only is able to detect SMA wires with a worse resolution.

**Figure 10:**
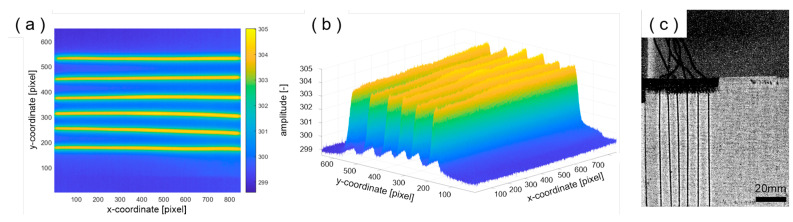
(**a**) Thermograms of a sample excited by electrical current, displayed in false-color representation of temperature. (**b**) 3D plot of the sample from (**a**), showing temperature corresponding to the depth position of the wires beneath the surface. (**c**) Grayscale image of the flash-excited thermography after Fourier transformation (*A* × ∗*ρ*/(180°)).

For thermography, the heat conductance of aluminum posed a challenge because of totally blurring the heat signature on the surface, so the composites were only tested without aluminum layers. Unfortunately, the application of active thermography using heat induced by eddy currents was unsuccessful. So, in the next step, intrinsic heating by applying electrical current was used for connectable wires. In this case, the wires were well localized in x- and y-coordinate direction (Figure 10a) as well as in depth (Figure 10b). Additionally, Figure 10b shows that the wires were not placed straight in the laminate but are slightly undulated though no wire breakage occurred. Besides these excitation methods, active pulse thermography with flash excitation was used. After a FFT analysis of the thermography images, calculated amplitude images clearly show the wires with a diameter of 250 µm (Figure 10c).

## 4. Discussion

In this paper, methods for monitoring SMA-wired GFRP composites are shown. After overcoming some difficulties in the production process, GFRP composite material with embedded SMA wires could be used for electrical and mechanical experiments. One difficulty lies in balancing the pressing force of the thermoforming process so as not to damage the wires on the one hand, and to avoid excessive overflow of the polymer on the other. Overflow must not prevent the wire ends from being connectable to the measuring circuit. Another optimization problem involves selecting the prestress or prestrain of the wires to use the SMA effect but to also keep them in place during thermoforming process. Wires with a smaller diameter of 100 µm would be beneficial in terms of structural interference of the bulk material, but this study shows they bear 16% less load and therefore break more easily, so only wires of 250 µm diameter could be used successfully. It is also shown that Young’s modulus of the GFRP composites with embedded SMA wires with a diameter of 250 µm is similar for various samples and is 30.8 GPa on average.

In this paper, a self-built way to monitor resistance changes up to 16 SMA wires whilst applying forces on the composite is shown. With the principle of multiple wires for monitoring it is possible to state where a force and with which amount it is applied. Relative change in resistance of the different wire sensors were shown. While force was applied on different indentation points, relative change in resistance of 0.0004 to 0.0021 *Δ*R/R_0 was measured. Wires next to the applied force point should sense the highest force compared to the others at first thought. Considering Euler–Bernoulli or plate theory, the depth where the wire is placed plays a crucial role, as well. Relative change in resistance signals show an indication of the position above or below the neutral axis because of their sign. It is shown that wires in the nearest of the indentation points indeed show the highest relative change in resistance.

Another outcome of this paper is the ability of different NDT-methods to detect the position and the state of SMA wires in a GFRP composite:

SMA wires can be characterized very well with thermography, and computer tomography. In thermography, excitation methods like flashlight or electrical current are best suited to detect the SMA wires with a diameter of 250 µm. The wires even do not need to be electrically connected. However, SMA wires in composites with a thin aluminum sheet as cover layer could not be detected with thermography due to the high reflection at the surface. Computer tomography clearly shows the positions of the wires with a diameter of 250 µm, no matter if the samples were with or without thin aluminum sheets as cover layers. Ultrasonic testing gives an idea of the SMA wire position but the theoretical lateral resolution of 300 µm could not be reached in this case.

In this study thermography is a fast and with regard to computer thermography cheap method to characterize thin NiTi-wires in a GFRP composite. With its temperature resolution of 0.05 K, thermography is not only able to detect thin SMA wires in a very short time, but can provide an image of the position and the condition within the GFRP composite.

## 5. Conclusions

The paper describes challenges in the production and testing process of SMA-wired GFRP composite laminates. The decisive boundary conditions for the forces and temperatures applied to the composite and the embedded wires are described and interpreted. So in the further course of this paper SMA wires with a diameter of 250 µm instead of 100 µm were used. In a differential scanning calorimetry (DSC) Austenite start temperature −0.56 °C, austenite finish temperature 21.85 °C, martensite start temperature 19.26 °C and martensite finish temperature −3.95 °C were measured.

NDT-methods like thermography and computer tomography proved well suited for characterization of the integrity, location and condition of the wires inside the composites after the thermoforming process.

In thermography, excitation methods like flashlight or electrical current are best suited to detect the SMA wires with a diameter of 250 µm. It is also shown that the SMA wires can act as sensors for applied forces on the composite. Depending on the amount of wires/sensors as well as the location and the amount of the applied force, the GFRP composites can be characterized according to their local behavior in an efficient way. While a probe tip exerts a force up to 1500× *g* at different indentation points, relative change in resistance of 0.0004 to 0.0021 ΔR/R_0 was measured.

In further works, SMA wires in GFRP composites can be tested under different environmental conditions, like different humidity and temperature. One could think of using machine learning with a predefined pattern of force application and resulting sensor signals, as well. It was not possible to use an underlying two dimensional model derived from Euler–Bernoulli or plate theory starting from the grid-based wire distribution because in the production process, the position and depth of the wires sufficiently change. Therefore, having methods to visualize the actual wire profile is eminent and could be a start for an underlying numerical model, e.g., by finite element simulation.

In situ tensile tests while computer tomography is performed should be performed, too, to monitor crack initiation and growth as well as damage development on the microscale to characterize the influence of the wires on the structure. In the future, SMA wires will be used to act as actuators in the given material system, similar to [28].

## Figures and Tables

**Table 1:** CT scanning measurement parameters.

Tube Voltage [kV]	Tube Current [µA]	Magnification	Voxel Size (μm)
120	100	1.1	35

**Table 2:** Measured resistance of all SMA wires in five samples in unit Ω.

Wire	1	2	3	4	5	6	7	8	9	10	11	12
Sample 1	3.81	.	3.91	4.46	4.44	4.34	3.92	4.44	4.25	.	4.13	3.96
Sample 2	.	3.8	.	4.18	3.67	.	.	3.78	3.64	4.04	.	.
Sample 3	4.03	.	3.9	3.63	3.85	.	4.23	.	.	4.07	.	.
Sample 4	3.99	4.08	4.43	4.37	.	.	3.79	4.56	3.86	3.78	3.64	3.69
Sample 5	4.01	3.91	.	3.96	4.16	3.88	4.01	4.3	4.07	.	.	3.87

## Data Availability

The original contributions presented in this study are included in the article. Further inquiries can be directed to the corresponding author.

## Abbreviations

The following abbreviations are used in this manuscript:

ADC	Analog to digital converter
FFT	Fast Fourier Transformation
GFRP	Glass fiber reinforced polymer
NDT	Non-destructive testing
NiTi	Nickel-Titan
PCB	Printed conductor board
SMA	Shape-Memory alloy
SME	Shape-Memory effect
